# Can we predict if patients with SLE will require more than one cycle of rituximab?

**DOI:** 10.1093/rheumatology/keab527

**Published:** 2021-07-08

**Authors:** Raquel Fernández González, Rym Abida, Eugeniu Gisca, Leila Duarte, David A Isenberg

**Affiliations:** 1 Internal Medicine Department, Hospital Universitario de Ourense, Ourense, España; 2 Internal Medicine Department, University Tunis El Manar, Faculty of Medicine of Tunis, Tunis, Tunisia; 3 Internal Medicine Department, Hospital Garcia de Orta, Almada, Portugal; 4 Internal Medicine Department – Medicina II, Centro Hospitalar Universitário de Lisboa-Norte, Hospital de Santa Maria, Lisboa, Portugal and; 5 Rheumatology department, Centre for Rheumatology Research, University College London, London, UK

**Keywords:** SLE, rituximab, repeated cycles

## Abstract

**Objective:**

To identify clinical and serological features that distinguish patients with SLE who require single as opposed to repeated rituximab (RTX) cycles.

**Methods:**

All 175 SLE patients followed up at University College Hospital from 2000 onwards were retrospectively reviewed. They were divided into a one-RTX-cycle group and a multiple-cycle group (2 or more cycles). Patients included had a follow-up of at least 3 years after their first RTX cycle, unless they needed a second infusion sooner.

**Results:**

A total of 131 patients were included; 44 (33.6%) received one cycle of RTX and 87 (66.4%) received two or more. The former were older at diagnosis (31.4 *vs* 21 years, *P* < 0.001) and at first RTX infusion (39.9 *vs* 29 years, *P* < 0.001). This group of patients had more organs/systems involved (*P* = 0.044), more leukopenia, lymphopenia and thrombocytopenia (*P* = 0.001, *P* < 0.0001 and *P* = 0.003, respectively) and lower C3 levels (*P* = 0.035). They also had fewer immunosuppressive drugs before RTX therapy compared with those who required multiple RTX cycles (*P* = 0.003). There was no statistical difference in either the clinical or serological response after the first RTX cycle between both groups. Furthermore, patients who had received more immunosuppressive treatments were more likely to require more than one cycle of RTX infusions (*P* = 0.007).

**Conclusions:**

RTX is an effective option for SLE patients with severe flares. Patients who received more immunosuppressive drugs were more likely to receive more than one set of RTX infusions. This suggests that RTX is best used for SLE patients with no history of refractory disease.

Rheumatology key messages

- We report on the use of rituximab infusions in patients with active SLE.
- Patients who only required one rituximab cycle did not have a history of refractory SLE.
- Response to the first rituximab cycle is not predictive of further infusions during follow-up in SLE.

## Introduction

SLE is a major autoimmune rheumatic disease characterized by an uncertain course and treatment response. Although the outcome for SLE patients has improved over the past 50 years, due to the use of immunosuppressive (IS) drugs, dialysis and kidney transplant [1, 2], it continues to have increased mortality and progression to end-stage renal disease [3]. Treatment also contributes to damage. For example, the use of glucocorticoids (dose >6 mg of prednisolone per day) has been linked to a higher risk of osteoporosis, cataract, and growth retardation in children [4].

A better understanding of the pathogenesis of SLE led to the introduction of B cell depletion (BCD) therapy in 2000, which eliminates pathogenic peripheral B cells and promotes the expansion of naive B cells [5].

Rituximab (RTX) is a chimeric mouse/human mAb against CD20, an antigen located on the surface of B cells. Its first approved use was in 1997 for non-Hodgkin’s lymphoma [6]. In 1999, it was first used to treat RA at University College London Hospital (UCLH) [7]. Subsequently, we gave it to our SLE patients in 2000 with promising results [8].

After showing utility in open-label trials [9, 10], two randomized trials (EXPLORER in non-renal SLE [11] and LUNAR [12] for renal lupus) were performed. Unfortunately, RTX did not achieve its primary end points. A recent meta-analysis hypothesized that the possible reasons for this failure included: the background therapy [notably, high-dose CSs and concomitant IS drugs (e.g. MMF)] and a different patient profile in the randomized trials compared with open-label trials (refractory patients were not enrolled in the former) [13].

In spite of these disappointing results, RTX is used widely in patients with SLE. It is recommended by the EULAR [14] and ACR [15] for LN and for other features by the British Society of Rheumatology [16]. Its use is also sanctioned by the National Health Service in England.

Besides its use in refractory SLE [17], three studies have shown the benefit of RTX treatment early in the disease [10, 18, 19]: Condon *et al.* [18] demonstrated that patients recently diagnosed with biopsy-confirmed LN and treated with RTX followed by MMF hardly ever required CSs; only 2 of their 50 patients needed oral CSs during 2-year follow-up. Similar results were reported by Gracia-Tello *et al.* [19], who treated 16 mostly non-renal SLE patients with RTX, followed by AZA plus HCQ within 3 months of diagnosis. This treatment was effective and steroid sparing.

Uncertainties remain about the consequences of treating SLE patients with RTX. Unlike patients with RA, for whom (in our department) we frequently use the drug on a regular basis (e.g. every 6 months), and given the unpredictable frequency of lupus flares, we use BCD on an as-required basis. After 20 years of experience of using this approach, we thought it was timely, (especially as we now have substantial long-term follow-up data) to distinguish SLE patients who have needed just one set of RTX infusions from those who have required multiple sets. In particular, we sought to identify any relatively simple clinical or serological markers that might identify those who are more likely to require multiple infusions.

## Methods

In this retrospective longitudinal analysis, we reviewed all 175 patients from the SLE cohort attending the Centre for Rheumatology at UCLH who were treated with RTX from 2000 to 2020 and collected data up to their last assessment. The RTX protocol consisted of 1 g infusions at day 1 and day 15. Both infusions form one RTX cycle. Each patient was also given 125 mg i.v. methylprednisolone on the same day they received the RTX. Until 2007, we gave two doses of cyclophosphamide (one day after RTX). Subsequently, we have used just one CYC infusion (750 mg), though some patients refused to have it at all.

We included patients who had been followed up for at least 3 years after their first RTX cycle, unless they needed a second one sooner. We therefore excluded patients who had only one cycle of RTX after 2017 and those lost to follow-up or deceased within the 3 years of their first and only RTX cycle. We also excluded patients who had an adverse reaction after the first cycle and those who had principally had RTX for an overlapping condition, e.g. APS.

Patient records and electronic data were systematically reviewed. We identified for each patient the age at disease onset, gender, ethnicity, autoantibody profile, overlapping diseases, the number of organs involved and the treatment received before RTX. We calculated disease duration until the RTX was first given, the delay between disease flare and RTX, the kinetics of anti-dsDNA antibodies, and complement C3, lymphocyte and neutrophil levels in the blood before and 6 months after the first cycle of RTX. BCD after the first cycle was also assessed where possible. BCD was defined by an absolute level of CD19 count of <0.005 × 10^9^ after treatment [20].

Disease activity was assessed using the global score (GS) conversion of the 2004 BILAG index whereby: A = 12; B = 8; C = 1; D/E = 0 [21].

Response to the first RTX cycle was assessed by comparing the BILAG GS, the anti-dsDNA antibody levels and the C3 levels before and 6–8 months after treatment. We defined an improvement in the GS as a decrease of at least 4 points (the result of going from a BILAG index of A to B in at least one organ/system). A decrease in anti-dsDNA antibody levels was considered to have occurred when they fell by at least 50% or normalized. An increase in C3 levels was defined as an increase of at least 25% or normalization.

The analyses were assessed using IBM SPSS statistics 22. χ^2^ tests were used to compare qualitative proportions. The Student’s *t* test was used to compare quantitative means if the data met the assumptions of normality; otherwise, non-parametric tests were used. Correlations between quantitative variables were assessed using the Pearson test or the Spearman test, depending on the assumptions of normality.

This study did not require a formal ethics approval, since it was regarded as an audit by the ethics committee at UCLH.

## Results

Among the 175 reviewed patients, 131 met the criteria set out in the Methods. Patients excluded were lost to follow-up (*n* = 17), had a single RTX cycle after 2017 (*n* = 21), presented with an infusion reaction after their first cycle (*n* = 4) or had RTX for an overlapping catastrophic APS (*n* = 2). During their follow-up, 44 (33.6%) patients received just one set of RTX infusions, and 87 (66.4%) received two or more [3.37 cycles on average (range 2–13)] (Table 1).

**Table 1:** Number of administrations of RTX in the cohort

Number of RTX cycles	Number of patients	(%)	(%)
1	44	33.6	33.6
2	39	29.8	66.4
3	20	15.3	
4	10	7.6	
5	7	5.3	
6	7	5.3	
7	1	0.8	
9	2	1.5	
13	1	0.8	

RTX: rituximab.

### Demographic characteristics

The mean age at SLE diagnosis in the 131 patients was 25.6 years (s.d. 12) and the mean age at first RTX infusion was 34.4 years (s.d. 12.8). Of the 131 patients, 123 were female (93.9%) and 8 were male (6.1%). Ethnically, 39.7% were Caucasian, 32.1% African Caribbean, 17.6% South Asian and 3.1% Chinese. There was a history of smoking in 29.8% of the patients. The overall disease duration in this cohort was 18.09 years (range 1–44). Follow-up after the first RTX infusion was 9.22 years on average (range 1–20) (7 patients had 2 sets of RTX within a follow-up duration of <3 years).

Statistical analysis showed that patients who needed repeated RTX cycles were significantly younger at diagnosis (21 *vs* 31.4 years, *P* < 0.001) and at first RTX infusion (29 *vs* 39.9 years, *P* < 0.001) than those who needed one single cycle (Table 2). There were no other statistical differences between the two groups in regards to their demographic characteristics (Table 2). Patients who had more than one cycle were followed up significantly longer (9.9 *vs* 7.8 years, *P* = 0.018) (Table 2).

**Table 2:** Characterization of the cohort—overall and within each group

	Total	RTX = 1	RTX > 1	*P*
*N*	131	44	87	
Females, *N* (%)	123 (93.9)	42 (95.5)	81 (93.1)	0.717
Ethnicity, *N* (%)				
Caucasian	52 (40)	18 (40.9)	34 (39.5)	0.840
African Caribbean	42 (32.3)	14 (31.8)	28 (32.6)	0.966
South Asian	23 (17.7)	8 (18.2)	15 (17.4)	0.894
Other	14 (10.7)	4 (9.1)	10 (11.5)	1.0
Age, mean (s.d.); (min; max)				
SLE diagnosis	25.88 (11.9); (6; 69)	31.4 (8.9); (13; 51)	23 (12.2); (6; 69)	<0.0001*
First Rituximab treatment	34.47 (12.8); (13; 73)	39.9 (10.9); (21; 64)	31 (12.8); (13; 73)	<0.0001*
Time SLE to Rituximab (in months) median (IQR); (min; max)	98 (119); (0; 456)	81 (125); (0; 456)	102 (114); (0; 357)	0.575
Follow-up since 1st Rituximab (in years), mean (s.d.); (min; max)	9.22 (4.6); (1; 20)	7.8 (4.8); (1; 19)	9.9 (4.3) (1; 20)	0.018*
Mortality				
Deaths, *N* (%)	8 (6.2)	5 (11.4)	3 (3.4)	0.115
Age at death: mean (s.d.); (min; max)	42.9 (15.2); (19; 56)	50.5 (6.9); (41; 56)	32.7 (18.7); (19; 54)	0.132

RTX = 1: one rituximab administration group; RTX > 1: more than one rituximab administration group; IQR: inter-quartile range; min: minimum; max: maximum.

### Serological markers and overlapping diseases

Among the whole group of 131 patients at diagnosis, 80.2% had raised anti-dsDNA antibodies and 67.2% low C3 levels; 55.7% of patients had positive anti-Ro, 52.7% anti-RNP, 36.6% anti-Smith and 21.4% anti-La antibodies; 43.5% of patients had at least one overlapping disease, the most frequent being Sjögren’s syndrome (*n* = 17, 13%), APS (*n* = 16, 12.2%), RA (*n* = 11, 8.4%) and hypothyroidism (*n* = 13, 9.9%).

No statistical differences were found between the two groups of patients in terms of their clinical and serological baseline markers or overlapping diseases (Table 3).

**Table 3:** Clinical and serological characteristics before the first rituximab cycle

		Total	RTX = 1	RTX > 1	*P*
*N*		131	44	87	
Serology at diagnosis, *N* (%)	Low C3	88 (67.2)	32 (72.7)	56 (64.4)	0.380
	High anti-dsDNA Ab	105 (80.2)	32 (72.7)	73 (83.9)	0.130
	Anti-Ro	73 (55.7)	27 (61.4)	46 (52.9)	0.356
	Anti-La	28 (21.4)	10 (22.7)	18 (20.7)	0.788
	Anti-Sm	48 (36.6)	16 (36.4)	32 (36.8)	0.963
	Anti-RNP	69 (52.7)	21 (47.7)	48 (55.2)	0.420
	Anticardiolipin (IgG± IgM)	12 (9.2)	3 (6.8)	9 (10.3)	0.748
Manifestations, *N* (%)					
Constitutional		115 (87.8)	41 (93.2)	74 (85.1)	0.180
Mucocutaneous		121 (92.4)	42 (95.5)	79 (90.8)	0.494
Rash		86 (65.6)	26 (59.1)	60 (68.9)	0.335
Photosensitivity		19 (14.5)	7 (15.9)	12 (13.8)	0.706
Alopecia		31 (23.7)	8 (18.2)	23 (26.4)	0.324
Oral ulcers		35 (26.7)	11 (25)	24 (27.6)	0.808
Musculoskeletal		122 (93.1)	42 (95.5)	80 (91.9)	0.717
Cardiorespiratory		57 (43.5)	25 (28.7)	32 (36.8)	0.029*
Kidney		78 (59.5)	29 (65.9)	49 (56.3)	0.291
CNS		30 (22.9)	10 (22.7)	20 (22.9)	0.973
Haematological		109 (83.2)	38 (86.4)	71 (81.6)	0.673
Vascular		33 (25.2)	8 (18.2)	25 (28.7)	0.231
Leukopenia		20 (15.3)	13 (29.5)	7 (8)	0.001*
Lymphopenia		45 (34.4)	26 (59.1)	19 (21.8)	**<0.0001**
Thrombocytopenia		21 (16)	13 (29.5)	8 (9.2)	0.003*
Number of organs involved, mean (s.d.)		5.08 (1.32)	5.41 (1.1)	4.92 (1.39)	**0.044**
BILAG score before Rituximab median (IQR) (min, max)		14 (10) (5; 56)	14 (5) (5; 56)	14 (10) (5; 46)	0.900
Serology before first RTX, mean (s.d.) or (min; max)	Anti-dsDNA (IU/ml)	778.44 (0; 6022)	881.63 (9; 6022)	717.17 (0; 5000)	0.239
	Low C3	50 (54%)	21 (22.8%)	29 (31.5%)	0.708
Drugs before Rituximab, *N* (%)	Immunosuppression	105 (80.2)	30 (68.2)	75 (86.2)	0.003*
	AZA	71 (54.2)	14 (31.8)	57 (65.5)	**<0.0001**
	Calcineurin inhibitors	30 (22.9)	6 (13.6)	24 (27.6)	0.073
	MMF	43 (32.8)	14 (31.8)	29 (33.3)	0.862
	MTX	35 (26.7)	6 (13.6)	29 (33.3)	0.016*
	Steroids	115 (87.8)	38 (86.4)	77 (88.5)	0.553
	Antimalarials	96 (73.3)	29 (65.9)	67 (77)	0.183
Drugs with Rituximab, *N* (%)	CYC	79 (60.3)	28 (63.6)	51 (58.6)	0.749

RTX = 1: one rituximab administration group; RTX > 1: more than one rituximab administration group; serology: considered positive values; low C3: value <0.7 g/l; high anti-dsDNA Ab: value >50 UI; Ab: antibodies; leukopenia: <4000/mcl leukocytes; lymphopenia: <1000/mcl lymphocytes; thrombocytopenia: <150 000/ul platelets; IQR: inter-quartile range; min: minimum; max: maximum.

### Disease activity and organ involvement

When RTX infusion was first indicated, patients had on average 5.08 cumulative organs/systems involved (s.d. 1.32). Musculoskeletal involvement was present in 93.1% of patients, mucocutaneous in 92.4%, constitutional in 87.8% and renal in 59.5%. Before the first RTX infusion, disease activity assessed by the BILAG GS was 14 on average (range 5–56), the mean anti-dsDNA antibody level was 778.44 IU/ml (normal <50 IU/ml) (range 0–6022), and the mean C3 level was 0.74 g/l (normal 0.9–1.8 g/l) (range 0.03–1.54). The mean lymphocyte count was 1.134 × 10^9^/l (range 0.25–4.39) and the mean neutrophil count was 4.672 × 10^9^/l (range 0.96–14.39). CD19 levels were 0.136 × 10^9^/l on average (range 0–1.089).

Our statistical analysis showed that patients who required a single infusion had more organs/systems involved before RTX therapy (5.41 *vs* 4.92, *P* = 0.044) but fewer cardiorespiratory manifestations (28.7% *vs* 36.8%, *P* = 0.029). Leukopenia, lymphopenia and thrombocytopenia were more frequent in the one RTX cycle group (*P* = 0.001, *P* < 0.0001 and *P* = 0.003, respectively) (Table 3).

There was no statistical difference between anti-dsDNA levels before the first RTX infusion between the groups, but patients who required a single cycle had significantly lower C3 levels before treatment (0.66 *vs* 0.79 g/l, *P* = 0.035) (Table 3).

### Specific treatment before RTX

Among our 131 patients, 129 (98.5%) had had other major medications prior to their first RTX infusion: HCQ in 73.3%, CSs in 87.8% (at a mean maximal dose of 11.9 mg/d) and at least one IS drug in 80.2% of cases. RTX was first prescribed after a mean disease duration of 8.61 years (range 0–38). Statistical analysis did not show any difference regarding the timing of the first RTX cycle (Table 2).

As shown in Table 3, patients with a history of IS intake before RTX treatment had significantly more RTX cycles (*P* = 0.003). A statistically higher risk of repeated RTX cycles was also noted for patients who had received AZA and MTX prior to RTX compared with MMF and CYC (*P* < 0.0001 and *P* < 0.016, respectively).

When comparing patients who had IS treatment before RTX with those who did not, we found that patients who had received IS therapy were more likely to require multiple sets of RTX infusions (*P* = 0.007, Table 4). Furthermore, regardless of the number of RTX cycles, we found that patients without previous IS had more active disease (BILAG GS 20.57 *vs* 15.25, *P* = 0.015), higher anti-dsDNA antibody levels (1212.93 *vs* 661.58, *P* = 0.01) and lower C3 levels (0.56 *vs* 0.78, *P* = 0.01). Response to RTX treatment was more frequently observed among patients who did not have IS treatment previously, but only the anti-dsDNA decrease was statistically significant (31.4% *vs* 60%, *P* = 0.037) (Table 4).

**Table 4:** Comparison between patients with and without history of IS treatment prior to rituximab

	Total (*N* = 128)	IS (*N* = 105)	No IS (*N* = 23)	*P*
One RTX cycle/multiple cycles (*N*)	44/84	30/75	14/9	0.007*
Number of organs involved, mean (s.d.)	5.05 (1.03)	5.16 (1.28)	4.52 (1.34)	0.033*
Before RTX infusion				
BILAG GS, mean (min; max)	16.28 (5; 56)	15.25 (5; 46)	20.57 (5; 56)	0.015*
Anti-dsDNA levels (IU/ml) mean (min; max)	755.31 (0; 6022)	661.58 (0; 6022)	1212.93 (28; 6000)	0.01*
C3 levels (g/l), mean (s.d.)	0.75 (0.3)	0.78 (0.3)	0.56 (0.25)	0.01*
Response to first Rituximab cycle				
Anti-dsDNA decrease, *N* (%)	31 (24.2)	22 (31.4)	9 (60%)	0.037*
C3 increase, *N* (%)	29 (22.7)	22 (57.9)	7 (87.5)	0.226
BILAG GS improvement? *N* (%)	91 (71.1)	73 (76.8%)	18 (85.7)	0.559
Follow-up since 1st Rituximab (in years), mean (s.d.); (min; max)	9.2 (4.6); (0; 20)	9.54 (4.5); (1; 20)	7.26 (3.5); (0; 16)	0.025*

GS: global score; IQR: inter-quartile range; IS: immunosuppressive; max: maximum; min: minimum; RTX = 1: one rituximab administration group; RTX >1: more than one rituximab administration group.

### Early use of RTX cycles

**Fig. 1:**
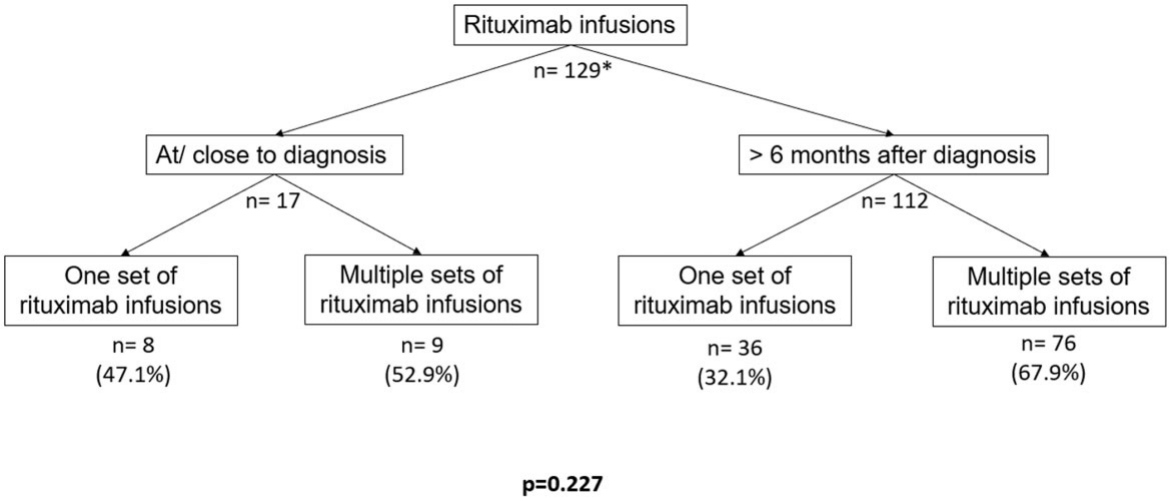
Number of rituximab infusions according to the time of disease diagnosis We divided our cohort in two groups: patients with early use of rituximab (those who first received rituximab within the first 6 months after diagnosis) and patients who first received rituximab after the first 6 months of the diagnosis. ^†^Two patients were not taken into account because of missing information regarding the date of disease onset.

Seventeen patients had received their first RTX cycle at or very close to the time of SLE diagnosis (13.2%); 52.9% of this17 patients required multiple infusions during their follow-up compared with 67.9% in the group of patients who had received their first RTX infusion after 6 months of diagnosis, but this difference was not statistically significant (*P* = 0.227) (Fig. 1).

### Response to the first RTX infusion

During the 6 months of follow-up after the first RTX cycle, GS improved by at least 4 points in 70.9% of cases, anti-dsDNA antibodies decreased by at least 50% or normalized in 23.7% of cases, and C3 levels increased by at least 25% or normalized in 22.9% of cases. The details for BCD in these patients are reported elsewhere [20].

There was no statistical difference between patients with a single RTX cycle and those with repeated RTX cycles in terms of the clinical or serological response to the first RTX cycle (Table 5).

**Table 5:** Response to the first rituximab infusion^a^

	*n* = 131 (all patients)	*n* = 44 (RTX = 1)	*n* = 87 (RTX > 1)	*P*
Total BILAG score, median (IQR) (min, max)	4.5 (6) (0; 25)	5 (6) (0; 17)	4 (6) (0; 25)	0.739
BILAG GS improvement, *N* (%)	93 (70.9)	33 (75)	60 (68.9)	0.483
C3 increase, *N* (%)	30 (22.9)	15 (34.1)	15 (17.2)	0.084
Anti-dsDNA decrease, *N* (%)	31 (23.7)	16 (36.4)	15 (17.2)	0.05

a RTX = 1: one rituximab administration group; RTX > 1: more than one rituximab administration group; IQR: inter-quartile range; min: minimum; max: maximum; GS: global score. At 6–8 months.

Our group of patients treated with only one cycle of RTX was not subsequently treated with belimumab or other mAbs (e.g. humanized antiCD20 mAbs), with just three exceptions: one infliximab, one ustekinumab and one belimumab.

### Mortality

In this cohort, eight patients (6.1%) died, five had one RTX cycle and three had two or more RTX cycles. The median age at death was 42.9 years (s.d. 15.2). The median time between the first RTX administration and death was 5.5 years (s.d. 1.52). All deceased patients had a history of class IV LN.

There was no difference in overall disease duration or the time since RTX between both groups (*P* = 0.10). We did not find any statistical differences in mortality between the two groups (Table 1).

## Discussion

The safety and effectiveness of RTX in SLE patients have been widely reported, in patients with refractory SLE and at an early stage of the disease. We have been using this treatment for two decades (22 years), with a mean follow-up of almost 10 years after the first infusion. We noticed that among patients who responded to the first set of RTX, some but not all subsequently required further RTX infusions during their disease course. We have attempted to ascertain whether there were any relatively simple clinical or serological differences that might distinguish these two groups.

In a multicentre observational study of 147 patients followed up for 13 months, Cassia *et al.* [22] compared the clinical and serological features of patients who received one set of RTX (*n* = 67) with those who had received a maintenance regimen (*n* = 80). This latter group was defined by at least three RTX courses with an interval of 4–8 months between infusions. Patients who responded to a single RTX cycle had fewer previous IS treatments and lower C4 levels (*P* = 0.034 and *P* = 0.008, respectively). The LESIMAB study was a multicentre retrospective study of 126 patients with refractory SLE, in which Fernández-Nebro *et al.* [23] compared the outcomes of single and repeated courses of RTX infusions. Patients who responded to one RTX course had more active disease and no history of severe haematologic flare.

Our cohort of 175 SLE patients treated with RTX is perhaps the largest, single-centre cohort with the longest follow-up to be reported [24]. Among the 131 patients studied in this report, we found that those requiring a single RTX course were older at diagnosis and at RTX infusion, had more organs/systems involved, had lower C3 levels and had received fewer previous IS drugs, especially less frequent use of AZA and MTX. Although patients with one RTX cycle had a shorter follow-up duration, these findings highlight previous conclusions suggesting that the best patients for this treatment might be those with more active disease and without a refractory history [22, 23].

The connection between clinical response and the degree and duration of BCD has broadly been established. Thus, complete BCD is associated with better outcome [1, 25], and the longer the duration of BCD the greater the benefit [1]. Rarely, complete BCD lasting years occurs after one single cycle of RTX [26]. B cell repopulation was not always followed by a flare [27], and a relapse might precede B cell repopulation [1]. Thus, BCD does not seem to be the best predictor.

Dias *et al.* [1] reviewed 98 patients followed up for 5 years, who were divided into two groups (one had B cell repopulation in the first year and the other did not). A short period to repopulation was associated with alopecia. A long duration until repopulation was strongly associated with lymphopenia and thrombocytopenia. Cambridge *et al.* [27] reviewed 25 patients to compare those who had a flare after 1 year of RTX with those with no relapse. They reported an association between anti-Ro/SSA and a short clinical response, but this correlation was not found with other serological markers or BAFF levels. Freitas *et al.* [20] reviewed 165 patients and concluded that patients with kidney involvement tend to fail treatment with RTX less often, as do those with higher disease activity and high anti-dsDNA antibody levels.

Patients with a single RTX infusion had a higher number of organs/systems involved prior to treatment (5.41 *vs* 4.92). There was no particular organ involved that could predict the number of RTX cycles except for the cardiorespiratory system. We would therefore assume that patients who received only one RTX cycle had a more florid flare with multiple systems involved, although we could not find any statistical difference in the initial BILAG GS between the two groups. It was reported in the LESIMAB trial [23] that the probability of a positive response to RTX increased by 10% with each additional point in the SELENA-SLEDAI index score and that it was seven times higher in patients who had required very high CS doses at one point.

Haematological involvement is frequent in SLE patients; sometimes there are several cytopenias refractory to the treatment [28]. In our cohort, more patients with leukopenia, lymphopenia and thrombocytopenia were found in the group of patients who received a single set of RTX infusions. This finding could be explained if the lymphopenia is due to an autoimmune mechanism, and there is a lengthy period of BCD post-RTX. Also, previous reviews show the effectiveness of RTX in SLE patients with thrombocytopenia [28].

Serologically, we did not find any association of receiving a single RTX course and anti-dsDNA, Sm, Ro, La or RNP antibody status or low complement at diagnosis. However, patients with lower C3 levels before RTX infusion were more likely to have a single RTX course during their follow-up.

Cambridge *et al.* [29] reviewed 16 SLE patients after RTX treatment and noticed a decrease in anti-nucleosome and anti-dsDNA antibody levels, with statistical significance after 6–8 months of follow-up. Similar results showing a decrease in anti-dsDNA antibody levels (and an increase in C3), after RTX treatment was reported in the EXPLORER trial [11]. In the present study, the anti-dsDNA antibody decrease 6 months after the first cycle of RTX was more frequently reported among patients with a single infusion compared with the multiple-infusion group (36.4% *vs* 17.2%), and this difference approached statistical significance (*P* = 0.05). No statistical difference was linked to the C3 increase (*P* = 0.084). Thus, according to our results, the serological response to the first cycle of RTX did not predict whether the patient would need more RTX infusions during the disease course.

Few previous reports have compared groups of patients according to the number of cycles of RTX given. In Cassia *et al.*’s cohort [22], the survival rate without relapse was similar between patients who received a single course and those on a maintenance RTX regimen; 35% of their patients did not experience any disease reactivation during maintenance treatment. This *sustained responders* group had a lower damage index at the time of the first RTX.

When we compared the delay between RTX infusions and disease onset, we found that half of the patients treated early with RTX required multiple sets, compared with two-thirds of those treated >6 months after diagnosis (Fig. 1). Although no statistical difference has been shown, this might suggest that early use of RTX reduces the requirement for multiple subsequent courses. Larger studies with longer follow-up are needed to confirm this assumption.

Interestingly, we found that patients with one set of infusions had fewer IS drugs prior to RTX treatment: 68.2% had received IS treatment before RTX therapy in the one-set group *vs* 86.2% in the multiple-set group. Statistical significance was particularly seen in the group of patients that received AZA and/or MTX. Inversely, we also found that patients who did not receive an IS therapy before RTX were more likely to require a single RTX course during their follow-up. This inverse association between the number of RTX cycles and the number of previous IS drugs was also reported by Cassia *et al.* [22], who found that patients who had fewer IS drugs previously responded better to RTX.

In our study, we have also shown that the response to treatment was clinically and serologically better in the group that did not previously receive IS drugs, even though only the anti-dsDNA antibodies decrease was statistically significant. Despite a statistical difference in the follow-up between the group who had previously received IS drugs and the group who had not, these findings suggest that RTX can be more effective in patients without a history of IS treatment. The influence of IS treatment on the efficacy of biologics is still debatable. Pirone *et al.* [30] reported that patients treated with MTX achieved a lower BILAG score 52 weeks after RTX treatment, suggesting a synergic effect for RTX-MTX. Further studies are needed to understand better the impact of IS treatment on RTX therapy.

The retrospective nature of our study limited the available data for patients at diagnosis and during their follow-up. We evaluated the response to the first RTX at 6–8 months, whereas we now know that patient response time to the infusion varies widely. To date, no accurate predictive factors identifying a delayed response have been found [24].

In conclusion, we have reported on the use of RTX infusions in patients with active lupus. Patients who required one set of RTX infusion did not have a history of refractory SLE and had received fewer IS drugs. This suggests that the sooner RTX is prescribed, the greater the likely long-term benefits. Early treatment with RTX can control disease activity, while limiting damage caused by CS and IS. Further studies should focus on the interaction between IS treatment and RTX. This should allow us to revise our treatment protocols and offer the best quality of life for our SLE patients.

*Funding:* No specific funding was received from any bodies in the public, commercial or not-for-profit sectors to carry out the work described in this article.

*Disclosure statement*: The authors have declared no conflicts of interest.

## Data availability statement

The datasets used and/or analysed during the current study are available from the corresponding author on reasonable request.
